# Corksorb Enhances Alkane Degradation by Hydrocarbonoclastic Bacteria

**DOI:** 10.3389/fmicb.2021.618270

**Published:** 2021-08-19

**Authors:** Valdo R. Martins, Carlos J. B. Freitas, A. Rita Castro, Rita M. Silva, Eduardo J. Gudiña, João C. Sequeira, Andreia F. Salvador, M. Alcina Pereira, Ana J. Cavaleiro

**Affiliations:** CEB – Centre of Biological Engineering, University of Minho, Braga, Portugal

**Keywords:** corksorb, alkanes, *Rhodococcus opacus*, *Alcanivorax borkumensis*, growth, bioremediation, biosorbent, comparative transcriptomics

## Abstract

Biosorbent materials are effective in the removal of spilled oil from water, but their effect on hydrocarbonoclastic bacteria is not known. Here, we show that corksorb, a cork-based biosorbent, enhances growth and alkane degradation by *Rhodococcus opacus* B4 (Ro) and *Alcanivorax borkumensis* SK2 (Ab). Ro and Ab degraded 96 ± 1% and 72 ± 2%, respectively, of a mixture of *n*-alkanes (2 g L^–1^) in the presence of corksorb. These values represent an increase of 6 and 24%, respectively, relative to the assays without corksorb. The biosorbent also increased the growth of Ab by 51%. However, no significant changes were detected in the expression of genes involved in alkane uptake and degradation in the presence of corksorb relative to the control without the biosorbent. Nevertheless, transcriptomics analysis revealed an increased expression of rRNA and tRNA coding genes, which confirms the higher metabolic activity of Ab in the presence of corksorb. The effect of corksorb is not related to the release of soluble stimulating compounds, but rather to the presence of the biosorbent, which was shown to be essential. Indeed, scanning electron microscopy images and downregulation of pili formation coding genes, which are involved in cell mobility, suggest that cell attachment on corksorb is a determinant for the improved activity. Furthermore, the existence of native alkane-degrading bacteria in corksorb was revealed, which may assist *in situ* bioremediation. Hence, the use of corksorb in marine oil spills may induce a combined effect of sorption and stimulated biodegradation, with high potential for enhancing *in situ* bioremediation processes.

## Introduction

Global oil demand averaged 10^8^ barrels per day in 2019 (U.S. EIA (Energy Information Administration), 2020), entailing intensive exploitation of petroleum resources and significant environmental risks. Accidental oil spills occur frequently in marine environments, causing severe damage to ecosystems and human population (Tornero and Hanke, 2016; Cruz et al., 2017; Daghio et al., 2017). Crude oil is mainly composed of hydrocarbons, in particular alkanes, that account for more than 50% of the hydrocarbon fraction. These compounds are relatively inert, originating severe ecological problems upon their release to the environment (TPHCWG (Total Petroleum Hydrocarbon Criteria Working Group), 1998; Singh et al., 2012).

In the oceans, although the average number of large- (>700 t) and medium-sized (7–700 t) oil spills caused by tankers has been progressively decreasing (ITOPF (International Tanker Owners Pollution Federation Limited), 2020), major accidents still occur at irregular periods (Tornero and Hanke, 2016). Several other sources also contribute for marine oil contamination, namely, drilling wastes and produced waters resulting from both onshore and offshore activities, oil well blowouts, releases from subsea equipment and pipelines, damages on oil platforms, and operational discharges in marine ports and harbors (Miola et al., 2009; Tornero and Hanke, 2016; Daghio et al., 2017).

Considering that most of the conventional remediation technologies are environmentally unfriendly (Lofrano et al., 2017) and that bioremediation may not be fast enough to prevent the severe damages caused by oil spills, immediate containment and physical removal of the oil guarantee a sufficiently fast and efficient response without significant environmental disturbances. Sorption is one of the most effective remediation techniques, and sorbent materials have demonstrated good results in removing hydrocarbons from contaminated sites (Doshi et al., 2018). One interesting approach is the use of biosorbents, which are materials from natural origin that are renewable and generally biodegradable. The use of agricultural or industrial wastes as biosorbents represents an effective, eco-friendly, and low-cost alternative that complies with the circular economy approach (Cojocaru et al., 2011; Doshi et al., 2018).

In particular, cork-based sorbents have been used for the removal of different pollutants, including hydrocarbons (Pintor et al., 2012, 2013), and a commercial product – Corksorb – is available for oil spill remediation (Martins and Reis, 2007). Corksorb presents an absorption capacity of up to 10 times its weight in oil (Pintor et al., 2012) and is obtained through thermal treatment of regranulated cork particles, which are by-products of cork stopper production (Pintor et al., 2013). Due to its particular physical and chemical properties, cork materials present good sorbent capacity and low water permeability (Pintor et al., 2013).

*In situ* or *ex situ* bioremediation of oil-contaminated environments/matrixes can be accomplished by hydrocarbonoclastic bacteria, which utilize the oil components as carbon and energy source for growth (McKew et al., 2007). Some of the most representative members of this group belong to the genera *Alcanivorax* and *Rhodococcus*. Members of both genera are able to produce surfactants, which is a relevant feature for hydrocarbon consumption (Rapp and Gabriel-Jürgens, 2003; Liu et al., 2010). *Alcanivorax* is considered the dominant Gram-negative genus degrading aliphatic hydrocarbons in saline environments. These bacteria have been applied in the bioremediation of oil-spilled marine ecosystems (Hassanshahian et al., 2014; Dashti et al., 2015; Gao et al., 2015) due to their capacity of using high hydrocarbon concentrations (specially alkanes up to C32) as sole carbon source (Yakimov et al., 1998; Schneiker et al., 2006; Al Kharusi et al., 2016). The genus *Rhodococcus* is one of the most versatile concerning hydrocarbon degradation, being able to metabolize different types of hydrocarbons – from alkanes with C6–C36 (Wentzel et al., 2007) to complex polycyclic aromatic compounds present in gasoline, diesel, engine, and crude oil (Koma et al., 2003; Binazadeh et al., 2009; Song et al., 2011; Auffret et al., 2015). They can be found in different natural environments, including marine sediments and water (Sharma and Pant, 2000; Heald et al., 2001; Peng et al., 2008). Due to its remarkable catabolic versatility, several works were performed using *Rhodococcus* strains in oil bioremediation strategies (Gargouri et al., 2011; de Carvalho, 2012; Suja et al., 2014).

In this work, we hypothesize that oil sorption by biosorbents can be combined with oil biodegradation by hydrocarbonoclastic bacteria, representing a novel approach for (bio)remediation of oil spills. Moreover, we hypothesize that the presence of the biosorbent may stimulate the activity of these bacteria, e.g., by promoting the contact between bacteria and hydrocarbons, acting as a support for bacterial growth or by containing stimulatory compounds (e.g., nutrients, cofactors) in its chemical composition, which could contribute to enhance oil spill bioremediation. The potential of corksorb to enhance growth and hydrocarbon degradation by hydrocarbonoclastic bacteria was investigated in batch assays. A mixture of *n*-alkanes, the main components of crude oil, was used as carbon and energy source. Two different bacterial strains were tested: *R. opacus* B4, a Gram-positive bacterium able to degrade both aromatic and aliphatic hydrocarbons (Na et al., 2005; Castro et al., 2016), and *A. borkumensis* SK2, a marine Gram-negative bacterium isolated from seawater/sediment samples that uses almost exclusively alkanes as carbon and energy source (Yakimov et al., 1998; Baik, 2010). Transcriptomics analysis of *A. borkumensis* SK2 growing on alkanes in the presence and absence of corksorb was also performed. Additionally, the presence of native bacteria in corksorb, capable of growing with alkanes, was investigated.

## Materials and Methods

### Biosorbent

Corksorb granules with particle diameters between 0.3 and 1 mm were provided by Corticeira Amorim, S.G.P.S. (Portugal). The corksorb granules may contain apolar hexane-extractable compounds that can potentially cause interferences in the identification and quantification of alkanes by gas chromatography (GC). To evaluate these potential interferences, triplicate assays were prepared in 250-mL Erlenmeyer flasks, each one containing corksorb (25 mg) and distilled water (50 mL). After incubation at 30°C in a rotary shaker (150 rpm) for 48 h, hydrocarbon extraction and quantification were performed. The presence of apolar hexane-extractable compounds in corksorb was confirmed (Supplementary Figure 1A), but the comparison of this chromatogram with the one from the alkanes’ mixture used in the assays (see section “Effect of Corksorb on Growth and Alkane Degradation by Hydrocarbonoclastic Bacteria”) (Supplementary Figure 1B) allowed to rule out relevant interferences of these compounds in the experiments performed in this work.

### Bacterial Strains and Growth Conditions

*Rhodococcus opacus* B4 (NBRC 108011) and *Alcanivorax borkumensis* SK2 (DSM 11573^T^) were purchased from the National Institute of Technology and Evaluation, Biological Resource Center, Japan (NBRC), and the Deutsche Sammlung von Mikroorganismen und Zellkulturen (DSMZ, Braunschweig, Germany), respectively. Maintenance and growth of the bacterial cultures were performed using 802 medium (NBRC, Supplementary Table 1) with agar (1.5%) for *R. opacus* B4 (Castro et al., 2016) and an artificial seawater mineral salts medium (ONR7a) (Supplementary Table 2) with agar and sodium pyruvate (10 g L^–1^) as carbon source for *A. borkumensis* SK2 (Dyksterhouse et al., 1995). Cultures were incubated at 30°C in a rotary shaker (120 rpm).

### Preparation of Seed Cultures

*Rhodococcus opacus* B4 was grown in solid medium for 4 days, after which a single colony was transferred to 50-mL mineral salt (MS) medium (Schlegel et al., 1961; Supplementary Table 3) supplemented with glucose (40 g L^–1^). A single colony of *A. borkumensis* SK2, grown in solid medium for 6 days, was transferred to 50 mL of ONR7a medium with 10 g L^–1^ sodium pyruvate. Seed cultures were grown in 250-mL Erlenmeyer flasks, at 30°C, with agitation (120 rpm) until reaching the middle of the exponential growth phase, i.e., 48 h for *R. opacus* B4 and 45 h for *A. borkumensis* SK2 (Supplementary Figure 2). Growth of the seed cultures was evaluated by optical density at 600 nm (OD_600_). Cells were harvested and washed two times with sterile phosphate buffered saline (PBS) solution (1×; Supplementary Table 4) by centrifugation at 13,000 min^–1^ for 10 min at 20°C. The pelleted cells of *R. opacus* B4 and *A. borkumensis* SK2 were then suspended in fresh MS and ONR7a media, respectively, and used as inoculum in the assays.

### Effect of Corksorb on Growth and Alkane Degradation by Hydrocarbonoclastic Bacteria

**FIGURE 1:**
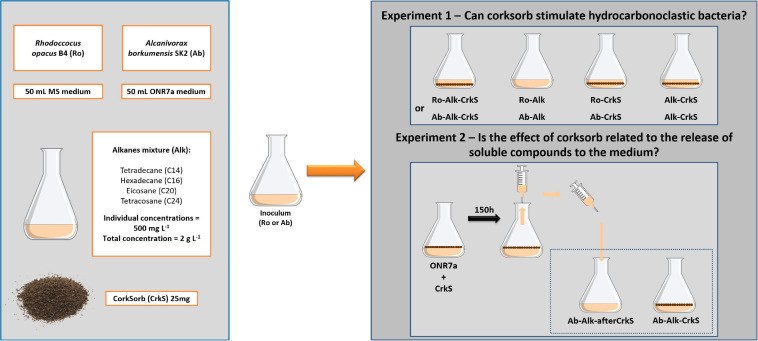
Scheme of the experimental procedure applied.

A mixture of *n*-alkanes was prepared in hexane, containing tetradecane (C14), hexadecane (C16), eicosane (C20), and tetracosane (C24), at individual concentrations of 50 g L^–1^. The assays were performed in sterile 250-mL Erlenmeyer flasks, containing corksorb (25 mg), a mixture of alkanes at a final total concentration of 2 g L^–1^ (corresponding to 500 mg L^–1^ of each individual alkane), sterile culture medium (50 mL), and the seed culture (*R. opacus* B4 or *A. borkumensis* SK2) at an initial OD_600_ of 0.2. The mixture of alkanes was added with a glass syringe under sterile conditions by using a Bunsen burner flame. Medium MS or ONR7a was used for *R. opacus* B4 or *A. borkumensis* SK2, respectively. These assays were designated Ab-Alk-CrkS and Ro-Alk-CrkS, as shown in Table 1 and Figure 1. Assays (i) without corksorb (Ab/Ro-Alk), (ii) without the mixture of alkanes (Ab/Ro-CrkS), and (iii) without inoculum (Alk-CrkS) were also prepared.

**TABLE 1:** Summary of the experimental conditions tested.

	Assay	Inoculum	Alkanes	Corksorb
Experiment 1	Ro-Alk-CrkS	✓	✓	✓
	Ro-Alk	✓	✓	x
	Ro-CrkS	✓	x	✓
	Ab-Alk-CrkS	✓	✓	✓
	Ab-Alk	✓	✓	x
	Ab-CrkS	✓	x	✓
	Alk-CrkS	x	✓	✓
Experiment 2	Ab-Alk-CrkS	✓	✓	✓
	Ab-Alk-afterCrkS	✓	✓	x*

Ro, *Rhodococcus opacus* B4; Ab, *Alcanivorax borkumensis* SK2; Alk, mixture of alkanes; CrkS, corksorb. * The medium was previously in contact with corksorb for 150 h, after which it was transferred (without corksorb) to new sterile flasks where the assays were performed.

All cultures were incubated at 30°C in a rotary shaker (120 rpm), and samples were collected over time after reaching the exponential growth phase (first sampling points at 283 h and 167 h for *R. opacus* B4 and *A. borkumensis* SK2, respectively). These values were defined based on preliminary tests carried out to evaluate the growth of the two hydrocarbonoclastic bacteria with the alkane mixture under study. For each sampling point, the total content of three replicate flasks was acidified at pH 2.0 with HCl (5 mol L^–1^) and preserved at 4°C until alkane analysis. The total content of the three other Erlenmeyer flasks was used for bacterial growth quantification. Growth was assessed by measuring total suspended nitrogen (TSN) due to the interference of alkanes on OD_600_ measurements. At the end of the experiment, samples of well-homogenized biomass were also collected from the assays containing corksorb and *A. borkumensis* SK2 (i.e., Ab-Alk-CrkS and Ab-CrkS), centrifuged at 10,000 *g* for 10 min at 4°C, washed with PBS, and stored at −20°C for further bacterial community analysis.

### Assessment of Potential Stimulation of *A. borkumensis* SK2 by Soluble Compounds From Corksorb

An experiment was designed to evaluate if the effect of corksorb on the growth of *A. borkumensis* SK2 was related to the release of soluble compounds into the culture medium. Erlenmeyer flasks containing only corksorb (25 mg) and medium ONR7a (50 mL) were incubated at room temperature for 150 h, without agitation, after which the medium was transferred using sterilized syringes to a new set of sterilized Erlenmeyer flasks. These were designated Ab-Alk-afterCrkS (Table 1 and Figure 1) and received also *A. borkumensis* SK2 seed culture and the mixture of *n*-alkanes, following the procedures described in section “Effect of Corksorb on Growth and Alkane Degradation by Hydrocarbonoclastic Bacteria”. Control assays containing fresh medium ONR7a, corksorb, alkanes, and *A. borkumensis* SK2 (Ab-Alk-CrkS; Table 1 and Figure 1) were also prepared as described in section “Effect of Corksorb on Growth and Alkane Degradation by Hydrocarbonoclastic Bacteria.” TSN was measured at the end of the assay using the total content of triplicate flasks.

### Effect of Corksorb on Differential Gene Expression and on Surface Tension of *A. borkumensis* SK2 Cultures

Erlenmeyer flasks of 1 L (total volume), containing 250 mL ONR7a medium and 125 mg of corksorb, were inoculated with *A. borkumensis* SK2. Control assays were performed without corksorb in order to compare the gene expression and surface tension (ST) when *A. borkumensis* SK2 was growing with and without corksorb. The alkane mixture and all the other experimental details were similar to the ones described in section “Effect of Corksorb on Growth and Alkane Degradation by Hydrocarbonoclastic Bacteria.” Samples from each culture (5 mL) were collected at different time points to evaluate biosurfactant production through ST measurement. For transcriptomics analysis, cell cultures (40 mL) were harvested at the exponential growth phase (167 h) and centrifuged (10,000 rpm, 10 min, 4°C), suspended in RNA later (Thermo Fisher Scientific) and frozen at −20°C until RNA extraction. Three independent cultures were sampled for each condition. At the same time point (*t* = 167 h), scanning electron microscopy (SEM) analysis was also performed.

### Enrichment Cultures

To evaluate the presence of native bacteria in corksorb and their capacity to grow using alkanes, enrichment cultures were developed. Corksorb (25 mg) was incubated in a sterile Erlenmeyer flask (250-mL volume) containing 50 mL of ONR7a medium at room temperature without agitation for 150 h. After this period, sterile loops were used to inoculate ONR7a agar (1.5%) medium with sodium pyruvate (10 g L^–1^). Incubation was performed at 30°C, 120 rpm. Growth was visually checked, and two sequential transfers were made to Erlenmeyer flasks with ONR7a medium (50 mL) using the mixture of *n*-alkanes described in section “Effect of Corksorb on Growth and Alkane Degradation by Hydrocarbonoclastic Bacteria” as carbon source at 1 g L^–1^ individual concentrations (total alkane concentration of 4 g L^–1^). The obtained enriched culture was designated CA (2) and characterized in terms of taxonomic composition.

### Analytical Methods

OD measurements were performed with a spectrophotometer U-1500 (Hitachi, Tokyo, Japan). TSN was quantified using standard cuvette tests (Hach-Lange GmbH, Düsseldorf, Germany) and a DR 2800 spectrophotometer after washing the samples three times with ultrapure water by centrifugation at 10,000 min^–1^ (15 min, 20°C). For alkane analysis, samples were sequentially extracted three times with hexane as organic solvent using separatory funnels, as described by Castro et al. (2016). Heptadecane (C17) was added as surrogate to the samples, at a final concentration of 200 mg L^–1^, from a stock solution prepared in hexane. The extracts were cleaned with Sep-Pak Florisil^®^ cartridges (Waters, Milford, MA, United States) and concentrated to a final volume of 1 mL in hexane using TurboVap^®^LV (Biotage, Uppsala, Sweden). Alkane quantification was made by GC with a flame ionization detector (GC-MS Varian^®^ 4000, Agilent, Santa Clara, CA, United States) and a VF1-ms column (Agilent, 30 m × 0.025 mm, Santa Clara, CA, United States). Helium was used as the carrier gas at 1 mL min^–1^. Detector and injector temperatures were 360 and 285°C, respectively. Column temperature was maintained at 60°C for 1 min and then increased up to 290°C at a temperature ramp of 8°C min^–1^. Undecane (C11) was used as GC internal standard at a concentration of 380 mg L^–1^ in hexane. ST was determined by the Ring method (Rodrigues et al., 2006), at room temperature (25°C), using a KRÜSS K6 Tensiometer (KRÜSS GmbH, Hamburg, Germany). All the measurements were performed in triplicate. SEM analysis was performed using an SEM FEI Nova 200 (FEG/SEM) equipment (FEI, Hillsboro, OR, United States) at SEMAT (University of Minho, Guimarães, Portugal). Samples for SEM images were prepared and processed as described by Salvador et al. (2017).

### DNA Extraction and Amplification for Bacterial Community Analysis

Bacterial community composition was assessed in biomass samples collected from the assays containing corksorb and *A. borkumensis* SK2 (see section “Effect of Corksorb on Growth and Alkane Degradation by Hydrocarbonoclastic Bacteria”). Total genomic DNA was extracted using a FastDNA SPIN Kit for Soil (MP Biomedicals LLC, Santa Ana, CA, United States) according to the manufacturer’s instructions. DNA amplification, Illumina libraries preparation, amplicon sequencing (Illumina MiSeq, Inc., San Diego, CA, United States), and bioinformatics analysis were performed by RTL Genomics (Lubbock, TX, United States), and the methodology is detailed elsewhere (Salvador et al., 2019). The amplicon primer set used was the 28 f/388 R (Handl et al., 2011; MacIntyre et al., 2015), targeting the bacterial 16S rRNA gene. Nucleotide sequences were submitted to the European Nucleotide Archive (ENA) under the study number PRJEB36602.

### Transcriptomics Analysis

RNA later was removed from *A. borkumensis* cells by centrifugation, and total RNA was extracted by using FastRNA Pro^TM^ Soil-Direct Kit (MP Biomedicals, Solon, OH, United States), in accordance with the manufacturer’s instructions. RNA extracts were sent in dry ice to Eurecat (Reus, Spain), where DNAse treatment (18068-015, Invitrogen), RNA quality assessment (using the Agilent TapeStation team and the Agilent High Sensitivity RNA ScreenTape Assay), libraries preparation, and sequencing were performed.

The sequencing libraries were created using the Illumina Stranded Total RNA Prep ligation with Ribo-Zero Plus (20040525, Illumina). The obtained cDNA libraries were quantified by microfluidic electrophoresis on Agilent’s TapeStation equipment and the Agilent DNA High Sensitivity ScreenTape kit. The length and concentration of each sample were determined, and quantification was performed with Qubit. Sequencing was performed in the NextSeq2000 sequencing system (Illumina), generating millions 2 × 76 pb reads per sample.

Initial bioinformatics data analysis was performed by Eurecat, which included mapping against a reference genome using HISAT2 (2.2.1), annotation and quantification of aligned reads with StringTie (2.1.4), and differential gene expression level comparison using DESeq2 R package (1.30.0). Samples were normalized by the Relative Log Expression (RLE) method, and expression levels were represented in counts per million (CPM). Genes were considered differentially expressed, upregulated or downregulated, if the difference in expression between conditions was at least two-fold, double or half expression levels, respectively, and if the adjusted *p*-value obtained for that gene was less than 0.05.

Additional bioinformatics analysis of *A. borkumensis* SK2 differentially expressed genes was performed to obtain extra functional information. For that purpose, initial gene IDs (locus tag) were converted to National Center for Biotechnology Information (NCBI) RefSeq protein IDs and UniProt IDs. By using the UniProt ID mapping web service, information from cross-reference databases and FASTA sequences were obtained. Protein sequences were then submitted to reCOGnizer^1^ that retrieved information from Clusters of Orthologous Groups of protein (COG), EuKaryotic Orthologous Groups (KOG), Conserved Domain Database (CDD), Pfam, NCBIfam, TIGRFAM, Protein Clusters, and Smart databases.

FASTQ files were submitted to the European Nucleotide Archive under the study accession number PRJEB46411.

## Results and Discussion

### Effect of Corksorb on Growth and Alkane Degradation by Hydrocarbonoclastic Bacteria

Addition of oxygen or nutrients, such as nitrogen and phosphorus (Pontes et al., 2013; Cerqueira et al., 2014; Safdari et al., 2018), as well as surfactants (Nakazawa et al., 2016), has been studied as biostimulation strategies to increase the activity of hydrocarbon-degrading bacteria. Considering the unique chemical composition, structure, and properties of corksorb (Pintor et al., 2012, 2013), we hypothesize that this biosorbent may have the potential to enhance the growth and hydrocarbon degradation by hydrocarbonoclastic bacteria, which was studied in this work. Alkanes were chosen as model compounds, since they are the main components of crude oil and their biodegradation has an important impact on oil removal and environmental cleanup (Head et al., 2006). The *n*-alkanes tested are characterized by low water solubility, low vapor pressure, and high boiling point (Supplementary Table 5; U.S. Environmental Protection Agency, 2016) and thus present a reduced tendency to dissolve or volatilize, generally forming a floating layer at the water surface.

In the assays performed with *R. opacus* B4, total alkane degradation reached 96 ± 1% in the presence of corksorb, which was 6% higher than without corksorb (90 ± 2%) (*p* < 1.21 × 10^–5^). This bacterium was able to efficiently degrade the mixture of alkanes, particularly C14, C16, and C20, as they were no longer detected in the culture medium since the first sampling point (283 h of incubation) either in the presence or absence of corksorb. On the other hand, corksorb exerted a positive effect on C24 degradation, the alkane with the highest chain length tested. C24 concentrations averaged 73 ± 21 mg L^–1^ and 194 ± 42 mg L^–1^ in the assays with and without corksorb, respectively, from 283 h until the end of the incubations (715 h). These values correspond to a 24% increase in C24 removal in the presence of corksorb.

Corksorb did not influence the growth of *R. opacus* B4 (Table 2) possibly due to the naturally high alkane-degrading efficiency of this bacterium and to the fact that, among the alkanes tested, only C24 was more difficult to degrade. TSN concentrations of 85 ± 3 mg L^–1^ and 81 ± 6 mg L^–1^ were measured at the end of the experiment in the presence and absence of corksorb, respectively (Figure 1), showing no statistical differences. In the assays with *R. opacus* B4 and corksorb (without the mixture of alkanes, Ro-CrkS), TSN concentration was always lower than 2 ± 0 mg L^–1^.

**TABLE 2:** Growth of *R. opacus* B4 from alkanes, expressed as total suspended nitrogen (TSN) concentration, with corksorb (Ro-Alk-CrkS) and without corksorb (Ro-Alk). Control assays without alkanes (Ro-Alk) are also shown.

Time (h)	Total suspended nitrogen (TSN) (mg L^–1^)		
	Ro-Alk-CrkS	Ro-Alk	Ro-CrkS
0	1.4	1.4	1.4
283	26.7 ± 5.4	41.8 ± 0.4	1.0 ± 0.2
474	58.6 ± 7.5	41.0 ± 2.5	1.2 ± 0.2
715	81.2 ± 5.8	84.5 ± 2.9	1.3 ± 0.3

*The results presented are the averages and standard deviations for triplicate assays.*

**FIGURE 2:**
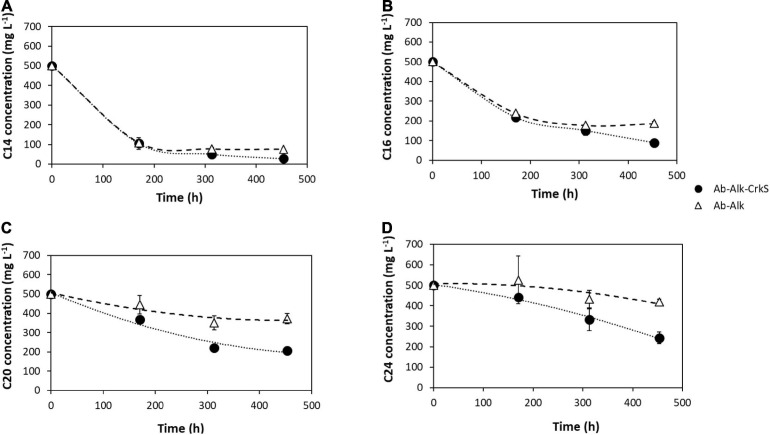
Alkane degradation by *A. borkumensis* SK2 with corksorb (Ab-Alk-CrkS, •) and without corksorb (Ab-Alk, △). **(A)** C14, **(B)** C16, **(C)** C20 and **(D)** C24. The results presented are the averages and standard deviations for triplicate assays.

In the assays with *A. borkumensis* SK2, significantly lower (*p* < 0.04) alkane concentrations were detected in the presence of corksorb, which corresponded to significantly higher degradation of all the alkanes tested when compared to the assays without corksorb (Figure 2 and Table 3). Total alkane degradation was 24% higher in the assays with corksorb relative to the assays without this biosorbent (i.e., 72 ± 2% and 47 ± 2%, respectively; Table 3). Degradation of longer-chain alkanes (C20, C24) was slower than that of C14 and C16 (Figure 2), confirming the more recalcitrant nature of longer-chain alkanes, thus requiring more time to be consumed, as reported by Head et al. (2006).

**TABLE 3:** Alkane degradation (%) by *A. borkumensis* SK2 at the end of the experiment, with corksorb (Ab-Alk-CrkS) and without corksorb (Ab-Alk).

	Ab-Alk-CrkS	Ab-Alk
C14	94 ± 2	85 ± 0
C16	82 ± 2	63 ± 2
C20	59 ± 4	26 ± 5
C24	51 ± 6	16 ± 3
Total	72 ± 2	47 ± 2

*The results presented are the averages and standard deviations for triplicate assays.*

The enhancement of alkane degradation by *A. borkumensis* SK2, induced by corksorb, was related to bacterial growth. After 452 h of incubation, the bacterial biomass (as TSN) was significantly higher (*p* < 0.02), i.e., 51%, in the presence than in the absence of corksorb, with TSN values of 12 ± 0 mg L^–1^ and 8 ± 0 mg L^–1^, respectively (Table 4). In the control assay Alk-CrkS, no growth was observed, and the expected alkane recovery was 98 ± 3% (based on previous experiments performed by Castro et al., 2017). All these results show that the higher alkane removal in the presence of corksorb is a result of alkane degradation and not an absorption phenomenon. Moreover, the results indicate that corksorb effectively stimulated *A. borkumensis* SK2 growth.

**TABLE 4:** Growth of *A. borkumensis* SK2 from alkanes, expressed as total suspended nitrogen (TSN) concentration, with corksorb (Ab-Alk-CrkS) and without corksorb (Ab-Alk). Control assays without alkanes (Ab-Alk) are also shown.

Time (h)	Total suspended nitrogen (TSN) (mg L^–^^1^)		
	Ab-Alk-CrkS	Ab-Alk	Ab-CrkS
0	4.7	4.7	4.7
167	4.4 ± 0.2	8.8 ± 1.1	3.5 ± 0.03
313	11.8 ± 0.2	8.4 ± 1.2	4.3 ± 0.6
453	12.2 ± 0.4	8.1 ± 0.4	5.6 ± 0.8

*The results presented are the averages and standard deviations for triplicate assays.*

Bacterial communities’ composition was analyzed in the assays with corksorb and *A. borkumensis* SK2 (i.e., Ab-Alk-CrkS and Ab-CrkS; Table 1 and Figure 1). *A. borkumensis* SK2 was the dominant genus identified both in the presence and absence of alkanes (Table 5), with relative abundances of 81 and 91%, respectively, based on 16S rRNA gene sequencing. Bacteria from the genera *Curtobacterium* and *Roseomonas* were also present in the assays with alkanes (Ab-Alk-CrkS; Table 5), accounting for 13 and 5% relative abundance, respectively. When *A. borkumensis* SK2 was incubated only with corksorb (Ab-CrkS), members of the *Rhizobiales* order were detected and represented 8% of the bacterial community, including *Pelagibacterium luteolum* (4% relative abundance) (Table 5).

**TABLE 5:** Bacterial community composition at the genus level in the assays Ab-Alk-CrkS and Ab-CrkS and in the enrichment culture CA(2).

Taxonomic identification at the genus level^1^	Relative abundance (%)			Closest cultured relatives^2^	Identity (%)^2^	Accession number
	Ab-Alk-CrkS	Ab-CrkS	CA(2)			
*Alcanivorax*	81	91	–	*Alcanivorax borkumensis* SK2	100.0	NR_074890.1
*Curtobacterium*	13	–	–	*Curtobacterium citreum* strain DSM 20528	100.0	NR_026156.1
*Roseomonas*	5	–	–	*Roseomonas deserti* strain M3	92.2	NR_159351.1
*Pelagibacterium*	–	4	–	*Pelagibacterium luteolum* strain 1_C16_27	100.0	NR_116053.1
Unclassified (*Rhizobiales*)	–	4	–	*Pelagibacterium halotolerans* B2	96.0	NR_102924.1
*Ochrobactrum*	–	–	49	*Ochrobactrum anthropi* ATCC 49188	100.0	NR_074243.1
*Gordonia*	–	–	43	*Gordonia aichiensis* strain E9028	100.0	NR_037030.1
*Pseudomonas*	–	–	4	*Pseudomonas alcaligenes* strain ATCC 14909	97.7	NR_114472.1
*Microbacterium*	–	–	3	*Microbacterium phyllosphaerae* strain P 369/06	98.7	NR_025405.1
Other^3^	2	1	2	–	–	–

*^1^Taxonomic identification at the genus level based on 16S rRNA genes sequences of 291 bp length by Illumina MiSeq. ^2^ Results of sequence alignment by using BLASTN toward the RefSeq_rna database. ^3^ Operational taxonomic unit (OTU) with relative abundance <1%.*

The ability to perform hydrocarbon degradation has been reported in bacteria from the *Curtobacterium* and *Roseomonas* genera (Bhattacharya et al., 2003; Zhao et al., 2011; Wedulo et al., 2014; Lumactud et al., 2016), although other strains within these genera do not have this ability, even when detected/isolated from petroleum-polluted sites (Rajaei et al., 2013; Subhash and Lee, 2018). In fact, the presence of bacterial strains that are not able to degrade hydrocarbons in oil-contaminated environments is quite common, generally ascribed to their tolerance to hydrocarbons or to their involvement in the degradation of intermediary compounds. *P. luteolum* type strain 1_C16_27^*T*^ was isolated from solid wastes of an oil shale chemical industry (Xu et al., 2011).

In the assays with corksorb, the presence of hydrocarbon-degrading bacteria, or other bacteria exhibiting tolerance to these compounds, suggest that they might have a role in the degradation of the added alkanes. Nevertheless, the high abundance of *A. borkumensis* SK2 in the microbial communities at the end of the assays points out that this bacterium was the key player in this process. Considering that *Alcanivorax* species tend to become the dominant group in bacterial communities present in marine waters contaminated with hydrocarbons (Cappello et al., 2007), the use of corksorb as a biosorbent material may represent an interesting strategy for enhancing its growth and *in situ* bioremediation.

### Assessment of Potential Stimulation of *A. borkumensis* SK2 by Soluble Compounds From Corksorb

The observed stimulatory effect of corksorb on the growth and alkane degradation in *A. borkumensis* SK2 may be associated with the presence of compounds (either soluble or embedded in the structure of corksorb) that can act as cofactors, stimulating the growth and activity of this bacterium. Therefore, in this experiment, the possible release of soluble compounds from corksorb to the medium was investigated. Growth of *A. borkumensis* SK2 was measured in experiments performed with medium that did not contain corksorb but had been previously in contact with it (Ab-Alk-afterCrkS assays; Table 1) and compared with the growth in the presence of the biosorbent (Ab-Alk-CrkS assays). Statistically higher growth (*p* < 0.006) was achieved when corksorb was present in the medium, reaching TSN values of approximately 30 ± 2 mg L^–1^ in the Ab-Alk-afterCrkS assays versus 24 ± 2 mg L^–1^ in the Ab-Alk-CrkS, after 300 h of incubation (Supplementary Figure 3). These results highlight that the presence of corksorb is essential, and that the release of soluble stimulatory compounds was not the main reason for the stimulation of *A. borkumensis* SK2.

### Effect of Corksorb on Differential Gene Expression and on Surface Tension of *A. borkumensis* SK2 Cultures

To further understand why *A. borkumensis* SK2 growth and alkane degradation are improved by corksorb, an additional experiment was performed. Corksorb may have a direct effect on alkane degradation or alternatively/additionally influence other cellular processes, such as biosurfactant production. Corksorb may also function as support for bacterial growth or promote the contact between the bacteria and the hydrocarbons (since these tend to float and bacteria are generally dispersed in the culture medium). Therefore, a transcriptomics analysis was performed to detect potential differences in gene expression. Particular focus was given to the genes involved in alkane degradation, biosurfactant production, and biofilm formation, which were described by Schneiker et al. (2006). Variations on ST were also assessed.

The Illumina sequencing originated a total of 905,664,672 reads that, after alignment with the reference genome, decreased to 383,563,992. A total of 2,825 different RNA molecules were detected. The results showed 128 upregulated genes and 130 downregulated genes when *A. borkumensis* SK2 was incubated with corksorb relative to the control without corksorb. A high number of differentially expressed genes (66) coding for hypothetical proteins were detected, but additional functional information could be obtained by performing conserved domain analysis with the reCOGnizer tool. The differentially expressed protein-coding genes could be assigned to the following COG functional categories: Cellular processes and signaling (77 genes), Information storage and processing (28 genes), Metabolism (64 genes), and Poorly characterized (28 genes). However, 61 genes, including 19 non-protein-coding genes, could not be assigned to any COG category (Supplementary Table 6). All the differentially expressed non-coding genes detected were found to be overexpressed in the presence of corksorb. These genes coded for rRNA or tRNA and are among the most upregulated genes detected in this experiment (Table 6; Supplementary Table 6). For instance, these genes were 3–27 times more expressed in the presence of corksorb (Table 6). These results corroborate the higher activity of *A. borkumensis* SK2 cells when growing with corksorb, as more rRNAs and tRNAs, involved in protein synthesis, were expressed. This is in agreement with the higher growth and alkane degradation by *A. borkumensis* SK2 observed in the presence of corksorb (Tables 3, 4; Figure 2). Nevertheless, few genes associated with alkane degradation were differentially expressed (Supplementary Table 6), namely, two alkane 1-monooxygenases (locus tag ABO_RS13835 and ABO_RS00610) and three other enzymes that are involved in alkane conversion to alkanol and alkanol conversion to alkanal (a ferredoxin reductase and two alcohol dehydrogenases, locus tag: ABO_RS01020, ABO_RS13850, and ABO_RS06065, respectively), that were downregulated in the presence of corksorb.

**TABLE 6:** List of upregulated genes, annotated as ribosomal RNA, transfer RNA, or related genes expressed by *Alcanivorax borkumensis* SK2 when growing in the presence of corksorb relative to the control assay without corksorb.

Gene identifier (Locus tag)	Number of times genes are upregulated	Annotation (NCBI RefSeq database)
ABO_RS14190	27	transfer-messenger RNA
ABO_RS14160	26	RNase P RNA component class A
ABO_RS02520	12	16S ribosomal RNA
ABO_RS10185	11	16S ribosomal RNA
ABO_RS01870	11	16S ribosomal RNA
ABO_RS14235	8	signal recognition particle sRNA small type
ABO_RS10180	8	23S ribosomal RNA
ABO_RS02525	7	23S ribosomal RNA
ABO_RS09450	7	tRNA-Pro
ABO_RS14330	6	6S RNA
ABO_RS01665	6	tRNA-Met
ABO_RS01885	6	23S ribosomal RNA
ABO_RS09645	6	tRNA-Met
ABO_RS07825	6	tRNA-Ala
ABO_RS07820	4	tRNA-Glu
ABO_RS06250	4	tRNA-His
ABO_RS04560	4	tRNA-Ser
ABO_RS09255	3	tRNA-Ser
ABO_RS01880	3	tRNA-Ala

**FIGURE 3:**
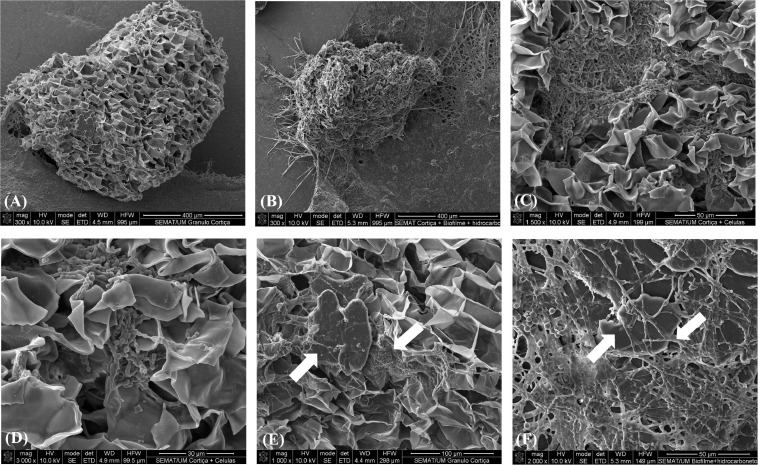
Scanning electron microscopy (SEM) images of **(A)** corksorb granule without *A. borkumensis* SK2, 400 μm; **(B)** corksorb granule surrounded by *A. borkumensis* SK2 biofilm, 400 μm; **(C)** *A. borkumensis* SK2 biofilm attached to corksorb, 50 μm; **(D)** *A. borkumensis* SK2 biofilm attached to corksorb, 30 μm; **(E)** detail of *A. borkumensis* SK2 biofilm (white arrow on the right) attached to corksorb in the vicinity of a carbonaceous *n*-alkane plate (white arrow on the left), 100 μm; and **(F)** *A. borkumensis* SK2 biofilm (white arrow on the left) attached to a carbonaceous *n*-alkane plate (white arrow on the right), 50 μm.

A total of 14 genes coding for different DUF domain-containing proteins were found, eight upregulated and six downregulated. However, these are poorly characterized domains and with unknown function, and therefore, their role in the increased growth and alkane degradation of *A. borkumensis* SK2 in the presence of corksorb remains unclear. Nevertheless, one should mention that the most downregulated gene during incubation with corksorb (32 times less expressed) is an uncharacterized protein containing a DUF1656 domain. Although the function is unknown, annotation against the Pfam database indicated that some DUF1656-containing proteins are putative membrane proteins (Supplementary Table 6). Several genes (a total of 15) related to pili formation (most Type IV pili) were downregulated by *A. borkumensis* SK2 when corksorb was present (Table 7). Pili are thin appendages in the surfaces of bacteria performing several functions, including twitching motility, uptake of external DNA to the cell, microcolony formation, biofilm formation, and adherence (Angelov et al., 2015; Craig et al., 2019). Probably because corksorb absorbs the hydrocarbons and offers a support for cell growth, some of the typical functions of pili were possibly less relevant for the bacteria growing with corksorb than when corksorb was not present. For instance, cells may have less necessity to move toward feeding. In this way, *A. borkumensis* SK2 population growing attached to corksorb probably shifts the energy toward cellular processes other than mobility. This hypothesis is reinforced by the SEM images obtained that show an extensive biofilm formed around corksorb granules (Figures 3B–D), as well as the proximity between hydrocarbons and cells (Figures 3E,F). This proximity could facilitate alkane degradation and consequently cell growth. Figure 3A shows a corksorb granule without biofilm, for comparative purposes.

**TABLE 7:** List of downregulated genes related to pili formation and respective functional annotation obtained when *Alcanivorax borkumensis* SK2 was incubated in the presence of corksorb relative to the control assay without corksorb.

Gene identifier (Locus tag)	Number of times genes are downregulated	Annotation against UniProt database	Annotation against COG and TIGRFAM databases
ABO_RS02430	12	UPI00031985B5: Uncharacterized protein; GspH/FimT family pseudopilin	COG4970: Type IV pilus assembly protein FimT
ABO_RS02420	5	Q0VSD3: Pilin biogenesis related protein	COG3419: Type IV pilus assembly protein, tip-associated adhesin PilY1
ABO_RS02400	5	Q0VSD7: Pilus biogenesis protein	COG4970: Type IV pilus assembly protein FimT
ABO_RS02425	5	UPI0005A1964E: Type IV pilin protein;secretion protein	COG4968: Type IV pilus assembly protein PilE
ABO_RS11470	4	Q0VMB6: Type IV fimbrial biogenesis protein PilP	COG3168: Type IV pilus assembly protein PilP
ABO_RS11485	4	Q0VMB3: Type IV fimbrial biogenesis protein PilM	COG4972: Type IV pilus assembly protein, ATPase PilM; TIGR01175: Type IV pilus assembly protein PilM
ABO_RS11480	4	Q0VMB4: Type IV pili biogenesis protein PilN	COG3166: Type IV pilus assembly protein PilN
ABO_RS01120	3	UPI0002E0144D: Type II secretion protein F	COG4965: Flp pilus assembly protein TadB
ABO_RS02405	3	Q0VSD6: Uncharacterized protein	COG4967: Type IV pilus assembly protein PilV; TIGR02523: Type IV pilus modification protein PilV
ABO_RS03175	3	Q0VRY5: Pilin protein family, putative	COG4969: Type IV pilus assembly protein, major pilin PilA
ABO_RS02410	3	UPI0003154941: Uncharacterized protein;N-terminal cleavage protein;PilW family protein	COG4966: Type IV pilus assembly protein PilW; TIGR02532: prepilin-type N-terminal cleavage/methylation domain
ABO_RS11475	2	UPI0015687014: Type IV pilus biogenesis protein PilO	COG3167: Type IV pilus assembly protein PilO
ABO_RS11465	2	Q0VMB7: Fimbrial assembly protein pilQ	COG4796: Type II secretory pathway, component HofQ; TIGR02515: Type IV pilus secretin (or competence protein) PilQ. Members of this family include PilQ itself, which is a component of the Type IV pilus structure, from a number of species.
ABO_RS01105	2	UPI0011D0765B: pilus assembly protein N-terminal domain-containing protein	COG4964: Flp pilus assembly protein, secretin CpaC
ABO_RS01110	2	Q0VT52: TadZ_N domain-containing protein	COG4963: Flp pilus assembly ATPase CpaE/TadZ, contains N-terminal REC/TadZ_N domain

Another possibility for the enhanced alkane degradation by *A. borkumensis* SK2 in the presence of corksorb could be a decrease in biosurfactant production due to the proximity between bacteria and hydrocarbons promoted by corksorb. This would potentially allow the bacteria to redirect their energy toward other cellular processes, such as growth. Biosurfactants have been demonstrated to efficiently emulsify hydrocarbons in several members of oil-degrading γ-Proteobacteria, facilitating hydrocarbon bioavailability, uptake, and biodegradation (Ron and Rosenberg, 2001). The biosurfactants produced by *A. borkumensis* SK2 are mainly glycolipids (Olivera et al., 2009). Here, we investigated if the genes coding for proteins involved in the production of glycolipid surfactants were differentially expressed by *A. borkumensis* SK2 in the presence of corksorb. Proteins involved in glycolipid surfactant production include glycoside hydrolases (also called glycosidases or glycosyl hydrolases), esterases, particularly lipases and also proteases and peptidases (Siebenhaller et al., 2018). We found three upregulated hydrolases (locus tag: ABO_RS05215, ABO_RS00720, and ABO_RS10845) and four downregulated hydrolases (locus tag: ABO_RS04970, ABO_RS05665, ABO_RS07690, and ABO_RS12680) in the presence of corksorb. Three different esterases were downregulated (ABO_RS03820, ABO_RS08725, and ABO_RS10855) and none was upregulated. Similarly, no lipases were upregulated, and one phospholipase was downregulated (locus tag: ABO_RS13795). No proteases were differentially expressed and no peptidases were upregulated, although five peptidases were found to be downregulated (locus tag: ABO_RS03130, ABO_RS03780, ABO_RS08845, ABO_RS08840, and ABO_RS05575). In addition, two glycosyl transferases (locus tag: ABO_RS04745 and ABO_RS04860) were downregulated when the culture grows with corksorb (Supplementary Table 6). However, none of these enzymes are specifically described as involved in surfactant production. Nevertheless, a gene coding for an outer membrane protein (locus tag: ABO_RS05955), that according to Schneiker et al. (2006) is possibly involved in emulsifier production, was found to be downregulated when *A. borkumensis* SK2 was incubated with corksorb. These results do not allow to conclude that the genes related to biosurfactant production are differentially expressed when corksorb is present.

**FIGURE 4:**
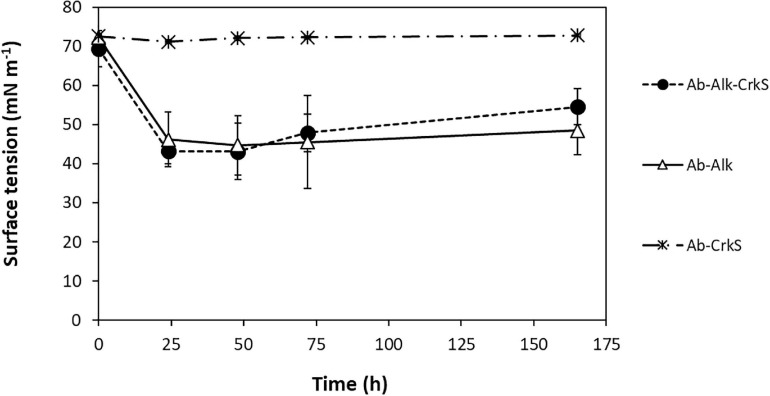
Evolution of surface tension (ST) over time in cultures of *A. borkumensis* SK2 grown with alkanes in the absence (Ab-Alk, △) and presence of corksorb (Ab-Alk-CrkS, •). Control assays without alkanes (Ab-Alk, *) are also shown. Results represent the average of three independent experiments ± standard deviation.

Also, the evolution of ST values, which is a direct measurement of biosurfactant activity, showed only minor differences between the cultures grown with and without corksorb (Figure 4). The efficiency of a surfactant is determined by its ability to reduce the ST (e.g., an effective surfactant can lower the ST of water from 72 to 30 mN m^–1^ (De et al., 2015). In our assays, the ST decreased to values of 43 ± 3 mN m^–1^ and 46 ± 7 mN m^–1^ in the presence and absence of corksorb, respectively, and these values remained approximately constant until the end of the experiment (Figure 4).

Transcriptomics and ST results show no evident relation between the improved activity of *A. borkumensis* SK2 in the presence of corksorb and the production of biosurfactants.

### Enrichment of Native Bacteria Present in Corksorb

Bacterial communities’ composition analysis performed with samples from the growth and biodegradation experiments (see section “Effect of Corksorb on Growth and Alkane Degradation by Hydrocarbonoclastic Bacteria”) showed the presence of native bacteria in corksorb that were capable of growing in the conditions tested. Different bacterial populations were also found in cork-processing wastewaters by del Castillo et al. (2012), which showed that some of the most prominent bacteria corresponded to well-known phenol-degrading organisms, namely, from the genera *Ralstonia*, *Stenotrophomonas*, *Cupriavidus*, and *Lysobacter*. Mesophilic isolates belonging to the genera *Klebsiella*, *Pseudomonas*, *Stenotrophomonas*, and *Burkholderia*, as well as thermophilic isolates of the *Bacillus* genus, were also obtained in enrichment cultures developed from cork boiling wastewater using tannic acid as a selective carbon source (Dias-Machado et al., 2006). If corksorb is used as a biosorbent in an oil spill scenario, the native bacteria from corksorb may be able to grow and contribute to hydrocarbon bioremediation, especially considering that the relative abundance of *A. borkumensis* at the moment of the oil spill is generally low.

Microbial growth could be visually noticed in the cultures developed from corksorb using the mixture of alkanes as carbon source (Supplementary Figure 4). The microbial community was dominated by *Ochrobactrum* and *Gordonia* species, with 49 and 43% relative abundance, respectively (Table 5). *Pseudomonas* and *Microbacterium* species were also present at lower relative abundances (i.e., 4 and 3%, respectively; Table 5).

Bacteria from *Ochrobactrum*, *Gordonia*, *Pseudomonas*, and *Microbacterium* genera that were found in the enrichment cultures are known for their capability to degrade hydrocarbons, and their presence in hydrocarbon-contaminated environments has also been reported (Franzetti et al., 2009; Arulazhagan and Vasudevan, 2011; Brzeszcz and Kaszycki, 2018; Trögl et al., 2018). However, these bacteria were not detected in the first experiment. The different conditions applied during the enrichment process, when compared to those previously used, can be the explanation for the differences observed in terms of bacterial community composition. These results reinforce the presence of native bacteria in corksorb, some of which are able to grow using hydrocarbons and thus might influence the positive effect of corksorb in alkane degradation.

In summary, this work shows that corksorb, currently used as oil-spill sorbent, herein contaminated with alkanes, can enhance bacterial growth and hydrocarbon biodegradation. The presence of corksorb was found to increase alkane degradation by *R. opacus* B4 and *A. borkumensis* SK2 (model hydrocarbon-degrading bacteria) in 6 and 24%, respectively, relative to the assays without corksorb. The growth of *A. borkumensis* SK2 was enhanced 1.51 times in the presence of corksorb. It was also found in this work that the positive effect of corksorb is not related to the release of soluble compounds. The increased expression of genes coding for rRNA and tRNA (required for protein synthesis) and the decreased expression of genes involved in pili formation (associated with several cellular processes, namely, cell motility), together with biofilm formation as revealed by SEM, can possibly explain the higher growth and alkane degradation by *A. borkumensis* SK2 in the presence of corksorb. Additionally, the native bacteria present in corksorb can possibly have a role in hydrocarbon biodegradation and may assist in *in situ* bioremediation of oil-contaminated environments.

Overall, the obtained results support a novel approach with potential to improve *in situ* marine bioremediation processes by coupling hydrocarbon sorption and biodegradation by hydrocarbonoclastic bacteria. This approach will generate lower amounts of oil-contaminated biosorbent than when only remediation by physical absorption is applied, thus representing a decrease in the costs associated with the treatment and regeneration of the oily biosorbent. Additionally, the oily biosorbent treatment can be performed *ex situ* by hydrocarbonoclastic bacteria that may convert the absorbed oil components into valuable compounds, such as lipids, biogas, or polyhydroxyalkanoates (Castro et al., 2017; Silva et al., 2021a,b).

## Data Availability Statement

The datasets presented in this study can be found in online repositories. The names of the repository/repositories and accession number(s) can be found below: https://www.ebi.ac.uk/ena, PRJEB36602 and https://www.ebi.ac.uk/ena, PRJEB46411.

## Author Contributions

ARC, MP, and AJC conceived this study. VM, CF, ARC, RS, EG, and AS performed the research. VM and ARC performed formal data analysis. JS and AS performed the bioinformatics data analysis. MP and AJC provided supervision, resources, and funding acquisition. VM and AJC drafted the manuscript. All authors contributed to the article and approved the submitted version.

## Conflict of Interest

The authors declare that the research was conducted in the absence of any commercial or financial relationships that could be construed as a potential conflict of interest.

## Publisher’s Note

All claims expressed in this article are solely those of the authors and do not necessarily represent those of their affiliated organizations, or those of the publisher, the editors and the reviewers. Any product that may be evaluated in this article, or claim that may be made by its manufacturer, is not guaranteed or endorsed by the publisher.

## Footnotes

1
https://bio.tools/recognizer
