# Supplementary material for: Corksorb Enhances Alkane Degradation by Hydrocarbonoclastic Bacteria
Source: Front Microbiol. 2021 Aug 19;12:618270. doi: 10.3389/fmicb.2021.618270 (PMC8417381; doi:10.3389/fmicb.2021.618270)
Supplement: Supplementary file 2 [file Data_Sheet_1.pdf]

Supplementary Material

1 Supplementary Figures

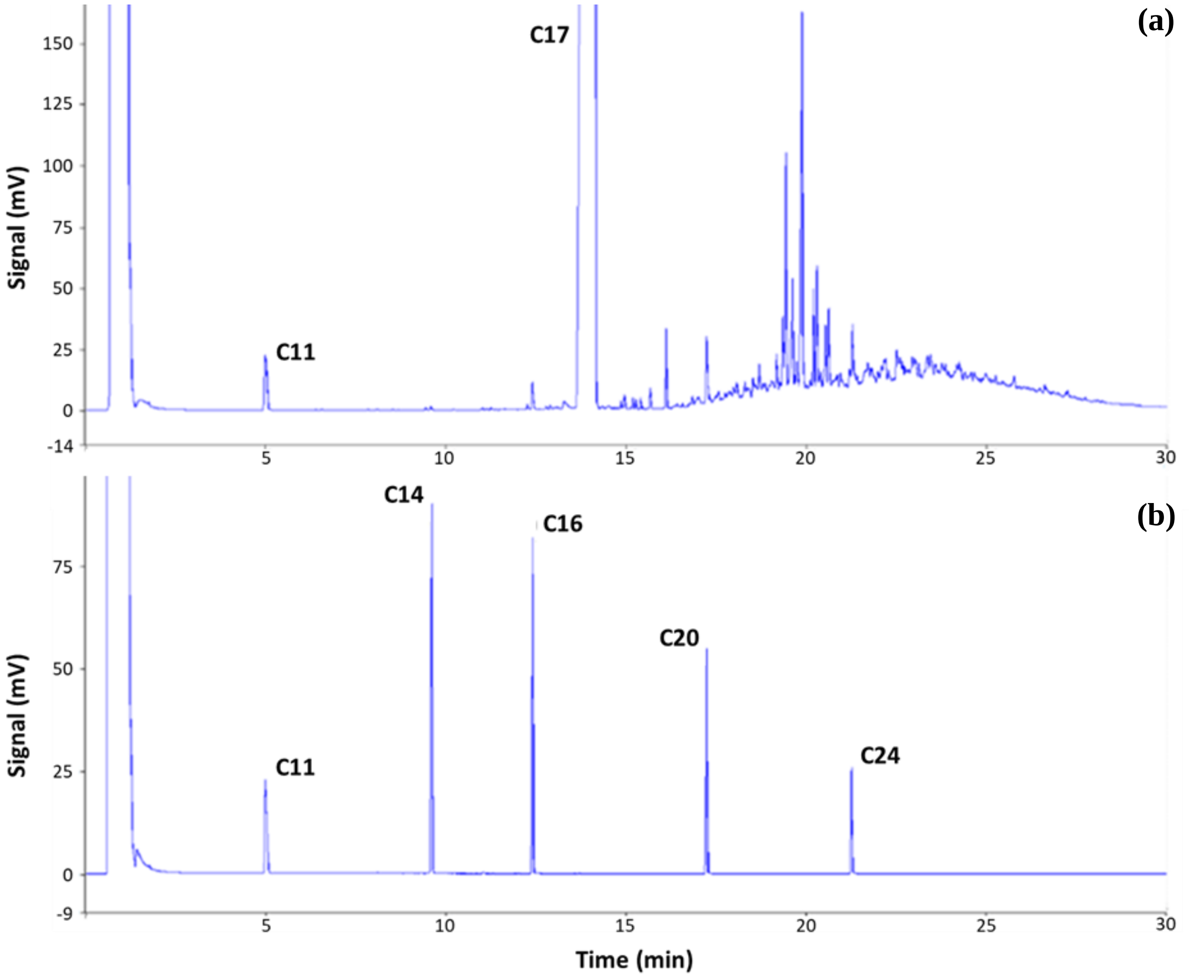

Supplementary Figure 1. Chromatogram of the compounds extracted from corksorb (50 times concentrated relatively to the assays) (a), and chromatogram of the alkanes mixture used in the assays, at 100 mg L^{-1} individual concentrations (5 times diluted relatively to the assays) (b). Undecane (C11) was used as internal standard, and its retention time is 5 min.

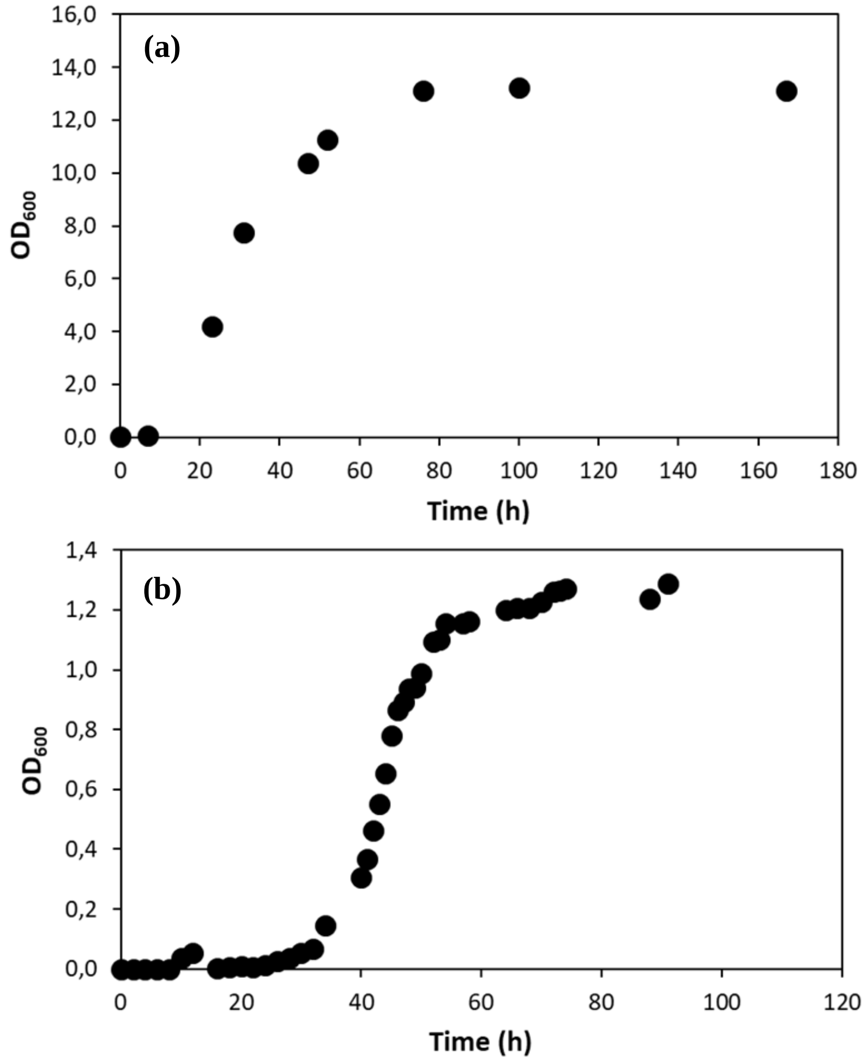

Supplementary Figure 2. Growth curves of *R. opacus* B4 in MS medium with glucose (40 g L^{-1}) (a), and *A. borkumensis* SK2 in ONR7a medium with pyruvate (10 g L^{-1}) (b).

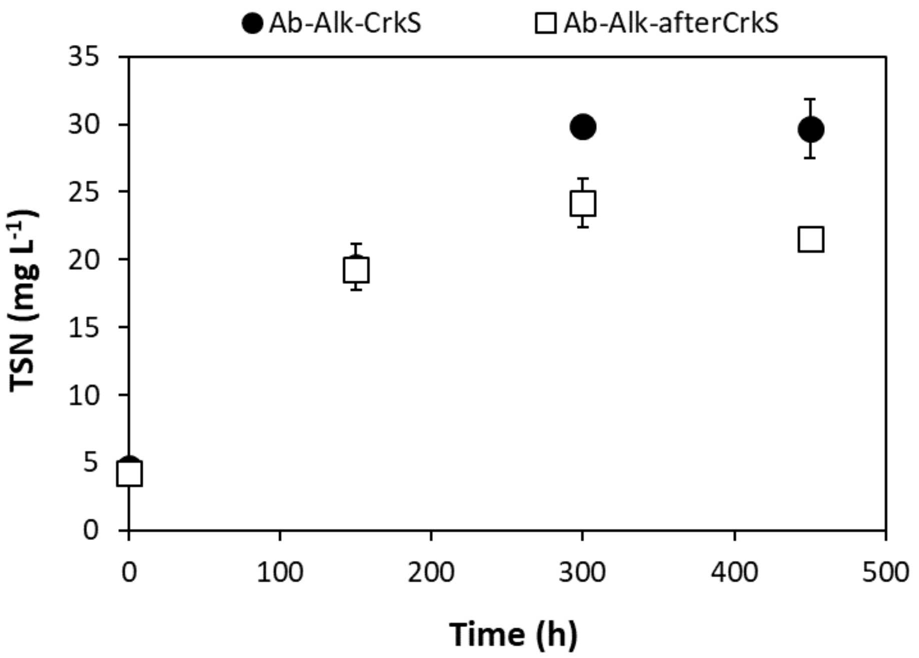

Supplementary Figure 3. Growth of *A. borkumensis* SK2 from alkanes, expressed as total suspended nitrogen (TSN) concentration, over time in the presence of corksorb (Ab-Alk-CrkS, ●) and in its absence (Ab-Alk-afterCrkS, □). In the assay performed without corksorb, the medium used was previously in contact with this biosorbent for 150 h, after which it was transferred to the assays flasks. The results presented are the averages and standard deviations for triplicate assays.

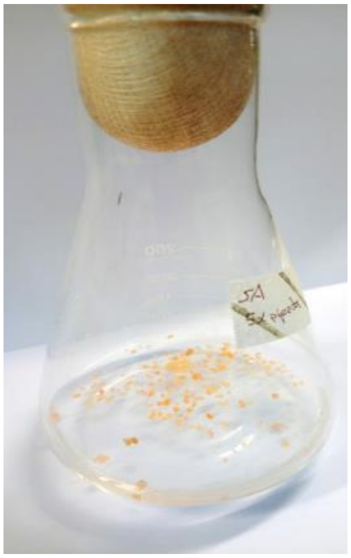

Supplementary Figure 4. Growth of native bacteria from corksorb in the medium with alkanes.

2 Supplementary Table

Supplementary Table 1. Composition of 802 medium (NBRC, Japan)

Component	Composition per liter
Polypeptone	10.0 g
Yeast extract	2.0 g
$MgSO_4$ x 7 H_2O	1.0 g

Supplementary Table 2. Composition of ONR7a medium (Dyksterhouse et al., 1995) – DSMZ medium 950 (DSMZ, Braunschweig, Germany)

Component	Composition per liter
NaCl	22.79 g
Na_2SO_4	3.98 g
KCl	0.72 g
NaBr	83.00 mg
$NaHCO_3$	31.00 mg
H_3BO_3	27.00 mg
NaF	2.60 mg
NH_4Cl	0.27 g
Na_2HPO_4 x 7 H_2O	89.00 mg
TAPSO	1.30 g
$MgCl_2$ x 6 H_2O	11.18 g
$CaCl_2$ x 2 H_2O	1.46 g
$SrCl_2$ x 6 H_2O	24.00 mg
$FeCl_2$ x 4 H_2O	2.00 mg

Supplementary Table 3. Composition of MS medium (Schlegel et al., 1961)

Component	Composition per liter
Na_2HPO_4 x 12 H_2O	9.0 g
KH_2PO_4	1.5 g
NH_4Cl	1.0 g
$MgSO_4$ x 7 H_2O	0.2 g
$FeNH_4$-citrate	1.2 g
$CaCl_2$	20.00 mg
Hoagland solution	2 mL
$NaHCO_3$	0.5 g

Supplementary Table 4. Composition of phosphate buffered saline (PBS) solution

Component	Composition per liter
NaCl	8.0 g
KCl	0.2 g
Na_2HPO_4 x 2 H_2O	1.44 g
KH_2PO_4	0.24 g
pH = 7.4	

Supplementary Table 5. Selected physicochemical properties of the *n*-alkanes tested (U.S. EPA, 2016)

**Property**	**C14**	**C16**	**C20**	**C24**
Molar mass (g mol^{-1})	198.4	226.5	282.6	338.7
Density (g cm^{-3})	0.756	0.760	0.762	0.760
Boiling point (°C)	253	287	281	391
Vapor pressure (Pa at 20 °C)	1.6	1.9×10^{-1}	6.2×10^{-4}	5.4×10^{-4}
Solubility in water (mol L^{-1} at 25 °C)	2.0×10^{-8}	4.0×10^{-9}	6.7×10^{-9}	7.3×10^{-9}

Supplementary Table 6. List of up- and down-regulated genes expressed by *Alcanivorax borkumensis* SK2, when growing in the presence of corksorb, with respective functional information retrieved from several databases (Uniprot, NCBI, COG, CDD, Pfam, NCBIfam, Protein Clusters, SMART, TIGRFAM) **[EXCEL FILE]**
